# Theoretical basis for the stabilization of charges by radicals on electrified polymers†

**DOI:** 10.1039/c6sc02672a

**Published:** 2016-11-23

**Authors:** Tomasz Mazur, Bartosz A. Grzybowski

**Affiliations:** a Institute for Basic Science, IBS Center for Soft and Living Matter, UNIST Ulsan 689-798 Republic of Korea grzybor72@unist.ac.kr; b Department of Chemistry, UNIST Ulsan 689-798 Republic of Korea

## Abstract

Quantum mechanical calculations at various levels of theory indicate that charges (both “+” and “−”) on organic polymers can be stabilized by radicals on nearby polymer chains. The stabilization mechanism is based on the formation of intermolecular odd-electron, two-center bonds with possible concomitant spin density redistribution (depending on the polymer and the number and type of proximal heteroatoms). This result is in line with our previous experimental demonstrations that on various types of polymers charged by contact electrification, radicals co-localize and help stabilize proximal charges (of either polarity). The principle of intramolecular charge-radical stabilization we now confirm on a fundamental level might have ramifications for the design of other macromolecular systems in which chemical reactivity is controlled by radicals flanking the charged groups or by charged groups flanking the radicals.

## Abstract 2

Quantum mechanical calculations at various levels of theory indicate that charges (both “+” and “−”) on organic polymers can be stabilized by radicals on nearby polymer chains.

## Graphical abstract

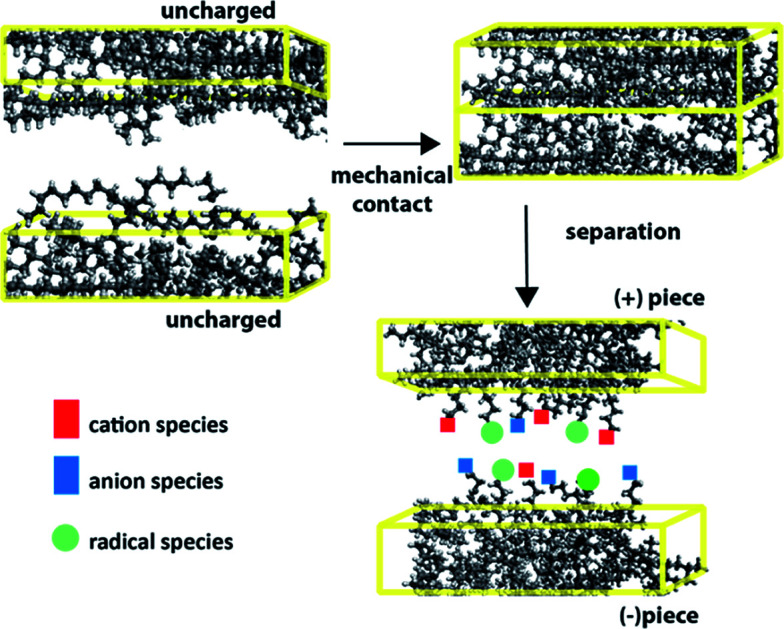

## 1. Introduction

**Fig. 1:**
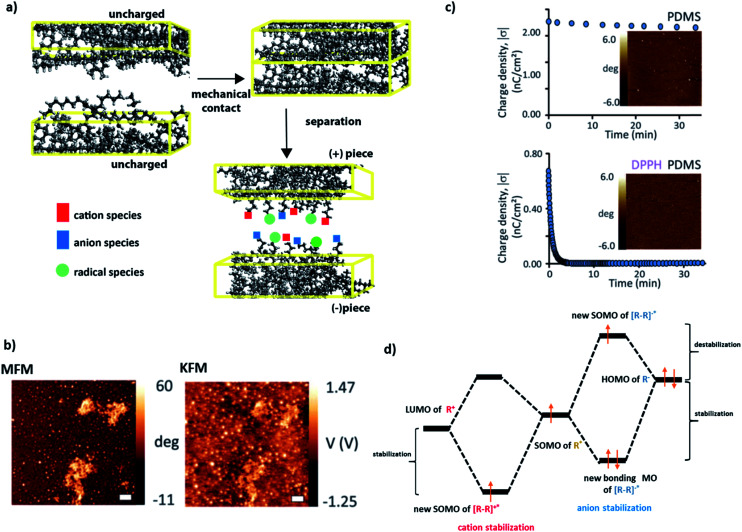
(a) During contact electrification, two pieces of polymers (different^37^ or identical^5^) are brought into contact and then separated with or without friction. In this process, polymer chains are broken, giving rise to either charged species (heterolytic scission: cations denoted by red squares, anions denoted by blue squares) or radical species (homolytic scission: radicals denoted by light green circles). (b) Kelvin Force Microscopy (KFM) and Magnetic Force Microscopy (MFM) imaging confirms that charges developed during contact electrification are co-localized with radicals (in this case, on the surface of Scotch tape). (c) Typical charge-decay curves for (left) pure PDMS contact-charged positively against polystyrene and (right) PDMS doped with 1 mM DPPH (2,2-diphenyl-1-picrylhydrazyl) radical scavenger and charged in the same way. The images in the insets give the corresponding MFM maps recorded after 24 hours. More radicals (white spots) are present on pure PDMS than on the rapidly discharging PDMS/DPPH. (d) Proposed qualitative molecular orbital diagrams illustrating stabilization of (left) cations and (right) anions by the nearby radical species with the stabilizing and destabilizing contributions indicated. Images in (b and c) reproduced from ref. 7.

It is well known in organic chemistry that highly-reactive radicals can be stabilized by adjacent functionalities within the same molecule *via* resonant effects/delocalization.^1–3^ A less explored question is whether and how radicals can interact with – and stabilize or be stabilized by – nearby molecules. A case in point here is our recent series of studies on contact electrification (CE) of polymers,^4–8^ during which heterolytic and homolytic bond breaking leads to the formation of, respectively, charged species and radicals (Fig. 1a). In particular, we showed^7^ that on such contact-charged surfaces, radicals co-localize with surface charges (Fig. 1b). Remarkably, when the radicals are removed (by small amounts of free-radical scavengers added to the polymer), the charges dissipate rapidly (Fig. 1c). For instance, addition of radical scavenging vitamin E renders various polymeric surfaces completely antistatic and capable of protecting electronic circuits from the effects of charge build-up and dielectric breakdown.

In ref. 7 we suggested that these effects might be due to the stabilization of the frontier orbitals of the charged polymer fragments (HOMO for anionic, LUMO for cationic) by the half-filled SOMO orbitals of the nearby radicals (Fig. 1d) – such an interaction could give rise to a new intermolecular bond comprising an odd number of electrons. Odd-electron bonds (one-electron two-center or three-electron two-center) have been proposed by Linus Pauling^9^ in the early 1930s and many examples have since been established (*e.g.*, gas phase H_2_^+^ or Li_2_^+^,^10^ molecules with phosphorous,^11^ carbon^12^ or sulphur^13,14^). In most of these examples, however, the bonds are short-lived and the instances where they would be longer-lived are relatively rare (*e.g.*, in some neutral species in solutions^15^ or for metal–ligand connections^16,17^). The unique feature of the charged polymer systems is that the charge-radical stabilization – and putative odd-electron bonding – persist for many hours (*cf.*Fig. 1c, upper panel).

The purpose of the present work is to verify whether the abovementioned scenario of stabilization and bonding is theoretically feasible at the quantitative level of requisite orbital overlap, symmetry, *etc.* To this end, we perform various types of quantum mechanical calculations on the models (short fragments) of polymers we had previously studied in the CE experiments – polyethylene (PE), polytetrafluoroethylene (PTFE) and polydimethylsiloxane (PDMS). These examples encompass species differing in the polarity of pendant bonds (PE *vs.* PTFE) and those differing in the composition of the backbone (all-carbon PE and PTFE *vs.* heteroatom-containing PDMS). We show that in all cases radicals can indeed stabilize charges on nearby molecules by several tens of kcal mol^−1^ (*i.e.*, much stronger than simple stabilization by van der Waals interactions, see ESI, Section 1†), form odd-electron bonds (in some cases with spin density redistribution), and yield thermodynamically stable secondary species. These results establish intermolecular charge-radical stabilization in contact-electrified polymers as a sound physical-organic principle which – in addition to purely fundamental interest – has important practical ramifications for the design of antistatic materials.

## 2. Computational methods

All calculations were performed using the Gaussian09 package.^18^ Unless noted otherwise, the 6-311++G(d,p) basis set was used for all atoms. Geometry optimization of the model systems was performed with the analytic gradients and Berny algorithms using the GEDIIS method. Various mutual orientations of the fragments were evaluated at the level of D3-UB3LYP/3-21G and the approximately “head-to-head” orientations minimizing the total energies, and simultaneously maximizing SOMO/LUMO or SOMO/HOMO overlap were chosen for further, more detailed analyses (see ESI, Sections 1 and 2†). Different levels of theory were considered, including Hartree–Fock, DFT with B3LYP xc potential and D3 method of Grimme.^19^ In order to correctly describe the singly occupied molecular orbitals (for radicals, cation–radicals and anion–radicals), all calculations for these species were performed in the spin unrestricted regime. Other species gave identical results for restricted and unrestricted regimes. No symmetry constraints were included for PE and PTFE (symmetry *C*_1_). For model systems accounting for cation rearrangements, transition states (TS) were optimized, and were distinguished from the stationary points by having only one imaginary vibrational frequency. From transition states, the total energy profiles were constructed with conformations of TS sampled by the QST3 algorithm. All ground states were tested for being the true minima by following vibrational frequency analysis and showed no imaginary frequencies. Throughout the manuscript, PE and PTFE systems are abbreviated according to the number of repeat units, *e.g.*, C2 corresponds to C_2_H_5_ species (cation, anion or radical). For the PDMS models, molecular geometries of species up to four repeat units were first optimized (with frequency analysis) at the D3-B3LYP/3-21G level of theory in the gas phase, and then single point calculations (with 6-311++G(d,p) basis set) were performed in order to obtain more exact energies. The electronic structure of PDMS-based systems was described with the incorporation of Natural Bond Orbitals (NBO ver. 6.0) technique of decomposition,^20^ which is more suitable for describing interaction of localized frontier orbitals of PDMS. Unless noted otherwise, orbital contours in all figures were plotted with the iso value of 0.02; for the spin-density contours, cutoff level of 0.004 was applied.

## 3. Results and discussion

### 3.1. Radical–cation stabilization

**Fig. 2:**
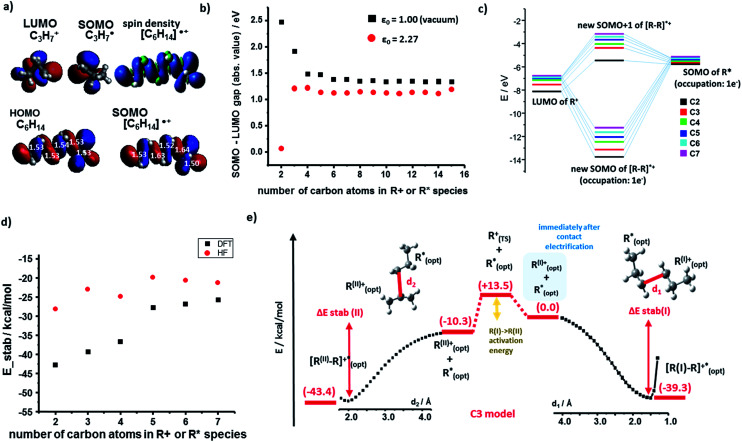
Cation–radical stabilization in polyethylene. (a) Optimized geometries and contours of the K–S HOMO (neutral molecule) and SOMO (charged species) molecular orbitals of the C3 fragments and their [C_6_H_14_]˙^+^ adduct. For [C_6_H_14_]˙^+^, spin density contour is also shown and compared to the HOMO of the uncharged C_6_H_14_. For the geometry optimization of the neutral molecule, optimized geometry of the radical cation species was chosen as a starting point. Numbers in white superimposed on the orbitals give the C–C bond lengths in Å. (b) The absolute values of the orbital energy gap in vacuum (*ε* = 1, black markers) and for the realistic dielectric constant of the polymer (*ε* = 2.27; red markers), both plotted as a function of the number of carbon atoms in each of the interacting fragments (here, the analysis is expanded up to 15 carbon atoms). (c) Molecular orbital energy diagram (in vacuum, dielectric constant *ε* = 1; only for alpha spins) for different sizes of model systems (from C2 to C7 fragments). Exact energies are listed in the ESI, Section 1.† (d) Total energy of stabilization calculated by DFT (black markers) and, additionally, by Hartree–Fock (HF, red markers) method. (e) “Reaction” total energy profile for the stabilization of a carbocation (here, C_3_H_7_^+^) by a radical (C_3_H_7_˙). The right portion of the plot is for the case where the primary cation cannot rearrange; in the left portion, this cation can rearrange into a more stable secondary cation – still, results do not change qualitatively. Similar energy profiles were also obtained for longer polymer fragments. Results in (d) and (e) are for the vacuum, *ε* = 1.

We begin by discussing the stabilization of cations by radicals. For the PE case, we studied systematically R^+^ and R˙ fragments up to seven monomers each. The SOMO (singly occupied molecular orbital) of R˙ and the LUMO (lowest unoccupied molecular orbital) of R^+^ can interact to form bonding, half-filled orbitals which we visualized and analyzed using the Kohn–Sham (K–S) approach.^21^ The spin density, *ρ*_(α)_ − *ρ*_(β)_ (providing information about localization of the unpaired electron) localized almost entirely in the SOMO of the [R–R]˙^+^species. Fig. 2a shows such representative density and SOMO for the [C3–C3]˙^+^ system. In these and other cases studied, the structure of the SOMO orbitals is very similar to that of the HOMOs of the corresponding neutral molecule (*e.g.*, C_6_H_14_ in Fig. 2a). This, in turn, indicates that the cation–radical interaction results, among others, in the formation of a molecular orbital (MO) of sigma (σ) symmetry, resembling a σ bond, though only half-filled. Another conclusion based on the SOMO [R–R]˙^+^ contours is that the unpaired electron is delocalized throughout the carbon chain – importantly, these results agree with classic experimental studies of Toriyama *et al.*^22^ who showed that *n*-alkane radical cations are characterized as delocalized σ radicals, with the unpaired electron delocalized over the entire chain and giving the characteristic 1 : 2 : 1, three-line ESR spectrum due to strong hyperfine couplings with the two in-plane end protons (ESI, Section 4 and S3 therein†).

The orbital interaction becomes more effective when the energy gap *i.e.*, absolute value of *E*(SOMO(R˙))−*E*(LUMO(R^+^)) decreases. We studied this difference using both vacuum calculations (black markers in Fig. 2b) and a more experimentally meaningful scenario of the dielectric constant of the medium approximating that of the polymer (*ε* = 2.25 for PE, in the simulations approximated by benzene solvent, *ε* = 2.27; data corresponding to red markers in Fig. 2d). As seen, for both scenarios, the magnitude of the gap decreases with increasing lengths of the interacting chains and approaches *ca.* 1 eV. Fig. 2c evidences that the orbital stabilization energies are favorable. Furthermore, we note that if the rearrangement of primary alkyl cations into secondary alkyl cations were to take place, this effect would only lower the energy gap (*cf.* energies for secondary R^+^ and [R–R]˙^+^ species listed in the ESI, Sections 1, and 3†).

Next, we calculated the total energies of stabilization (*i.e.*, difference between [R–R]˙^+^ complex and isolated fragments R^+^ and R˙, all in their optimized geometries) and the energetic profiles characterizing the approach of the two fragments (along a coordinate measuring the distance between terminal carbon atoms in the two fragments). The total stabilization energies in Fig. 2d calculated by either DFT or HF are all negative (also see ESI, Section 1,†) with the absolute DFT values relatively close to half of the dissociation energy of single carbon–carbon covalent bond in linear molecules (82.70 kcal mol^−1^/2 = 41.35 kcal mol^−1^) – this is, in line with the qualitative MO explanation above, whereby the highest occupied orbital of [R–R]˙^+^ is also a half-filled SOMO.

Fig. 2e shows full stabilization paths (with or without rearrangement of primary into secondary carbocations) calculated for the C3 system. The transition state (TS) was found only for the cation rearrangement step (not shown) and was not observed for the process of cation (primary or secondary)-radical stabilization. Lack of the TS implies negative activation energy (most likely of entropic origin), characteristic of very energetic species.^23–25^ Overall, the charge-radical stabilization process can be treated as a barrier-less reaction.^26^

**Fig. 3:**
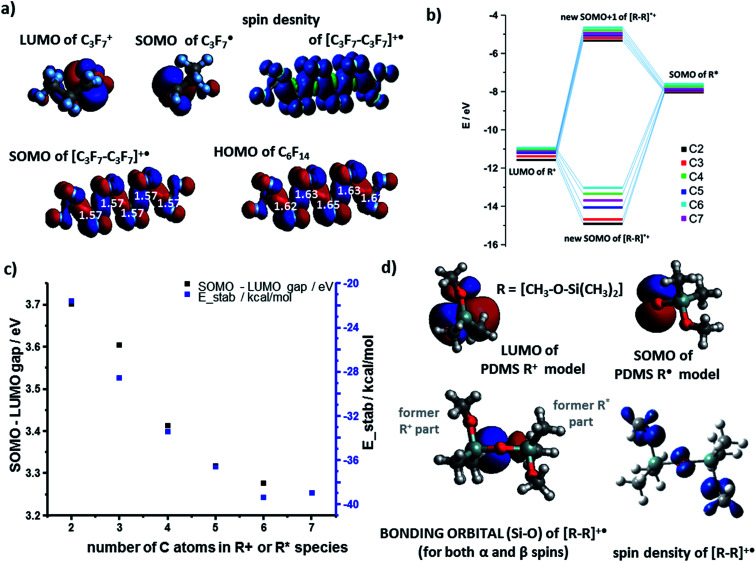
Cation–radical stabilization in (a–c) PTFE and (d) PDMS. (a) SOMO and LUMO orbitals of the C3 fragments making up the [C_6_F_14_]˙^+^ radical–cation adduct for which the spin density contour is also shown. HOMO of the uncharged C_6_F_14_. In white font – C–C bond lengths in Å. (b) Molecular orbital energy diagram (in vacuum, dielectric constant *ε* = 1; only for alpha spins) for different sizes of model systems (from C2 to C7 fragments). (c) Black markers and left axis: orbital energy gap plotted as a function of the number of carbons in each of the interacting fragments. Blue markers and right axis: total DFT stabilization energy plotted as a function of fragment size. (d) PDMS LUMO NBO contour of R^+^ and SOMO NBO contour of R˙ for 1-monomer model systems. NBO bonding orbital contour for [R–R]˙^+^ alongside with a spin density contour.

The results for the cation–radical species of PTFE and PDMS were generally similar to those of PE. For example, the SOMO orbital of the carbocation adducts resembled the HOMO of the neutral molecule (*e.g.*, [C_6_F_14_]˙^+^*vs.* C_6_F_14_ in Fig. 3a). The SOMO was, as before, of the bonding nature (Fig. 3b) though the *E*(SOMO(R˙))−*E*(LUMO(R^+^)) energy gap was wider than for the PE case (compare black markers in Fig. 3c and values in Fig. 2d). The total energies of stabilization were all negative and thus favoring formation of cation–radical species. The absolute values of these energies ranged from 21.9 kcal mol^−1^ to 38.9 kcal mol^−1^ (Fig. 3c, blue markers), which is somewhat less than for PE, and reflects the fact that SOMO electron density on highly electronegative F atoms is increased compared to the H's in PE, effectively weakening the carbon–carbon interaction in the radical–cation.

The case of PDMS is somewhat more involved as the differences in the electronegativity (1.9 for Si *vs.* 3.5 for O on the Pauling scale) of the backbone atoms must be taken into account. In our model, we assumed that anionic fragments can be terminated in O, cationic in Si, and radicals in either of these two atoms. Fig. 3d shows the LUMO(R^+^) and SOMO(R˙) NBO orbital contours of one-monomer PDMS fragments – these orbitals are centered on Si and O atoms, respectively. The shape of the resulting bonding orbital (for both α and β spins) is different from the spin density contour, indicating that the β electron density on the bonding orbital was transferred from nearby O atoms. The energy gap of the cation–radical adducts is 2.55 eV for this pair but increases to 3.43 eV for interactions between two-monomer fragments. The corresponding total energies of stabilization calculated by DFT are favorable: 66.8 kcal mol^−1^ and 74.5 kcal mol^−1^ respectively. The results were similar for systems of four monomers (longer chains were not studied due to many possible conformational minima one would have to consider).

### 3.2. Radical–anion stabilization

**Fig. 4:**
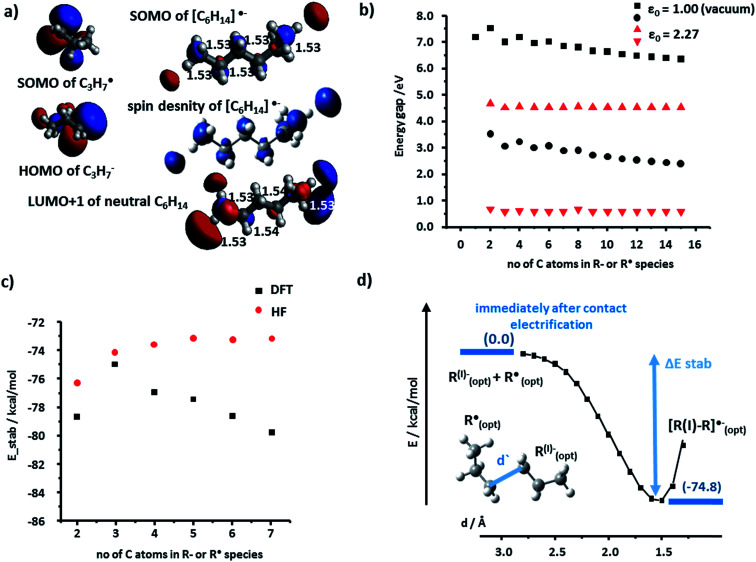
Anion–radical stabilization in polyethylene. (a) SOMO contour of the radical C3 fragment, HOMO of the anionic C3 fragment, and the SOMO and spin-density contours of the [C_6_H_14_]˙^−^ adduct. For comparison, the LUMO+1 of a neutral C_6_H_14_ molecule is also shown. In white and black fonts – C–C bond lengths in Å. (b) The absolute values of the orbital energy gap calculated in vacuum (*ε* = 1; black markers – rectangles for α spins and circles for β spins) and in a *ε* = 2.27 medium (red markers – triangles pointing up for α spins and triangles pointing down for β spins) plotted as a function of the number of carbon atoms in each of the interacting fragments (here, the analysis is extended to 15 carbon atoms in each chain). (c) Total energy of stabilization calculated by DFT (black markers) and by Hartree–Fock (HF, red markers) (d) “Reaction's” energy profile for the stabilization of a carboanion (here, C_3_H_7_^−^) by a radical (C_3_H_7_˙).

Heterolytic bond cleavage during contact electrification gives rise not only to cationic but also to anionic species – in fact, we observed experimentally both (+) and (−) regions on contact-electrified polymers (see ref. 4 and Fig. 1). To investigate stabilization (or its lack) during interactions of anions and radicals, we first considered the energy gaps between the interacting frontier K–S orbitals involved in the formation of [R–R]˙^−^ adducts. Following the same methods as in the case of cation–radicals, we determined that the spin density was localized on the SOMO whose structure was similar to that of the lower unoccupied orbitals (*i.e.*, SOMO is mostly the combination of LUMO or LUMO+1) of the neutral molecule R–R (see Fig. 4a for the C3 model). The orbital energy gaps (separately for the α and the β spins since the calculations were conducted in the unrestricted regime) for the PE radical–anion interaction were slightly over 6 eV (α spins) and 2.5 eV (β spins) (Fig. 4b, black markers). These values were smaller in calculations approximating the dielectric constant of the polymer (red markers in Fig. 4b; compare also with Fig. 2d). The SOMO of R˙ and the HOMO of R^−^ orbitals could interact to form filled bonding and half-filled antibonding orbitals, contributing to the overall stabilization energies on the order of 80 kcal mol^−1^. We note that when diffuse basis set was incorporated, the SOMO electron became localized outside of the molecule indicating the instability of the temporary anion.^27–30^ Such a spontaneous detachment of an excessive electron (or negative ion) from a molecular anion or anion radical is well documented^31–33^ and the electron can then stabilize in the neighboring defects.^34,35^

**Fig. 5:**
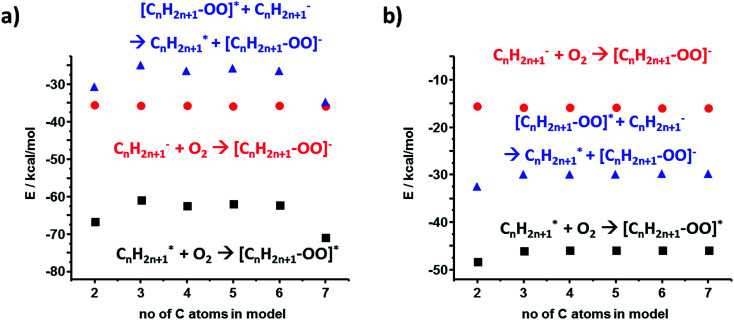
Calculations for an alternative scenario of anion–radical stabilization in PE mediated by the initial formation of superoxide radicals. Values in (a) are from DFT calculations, those in (b) are from HF.

For the polyethylene case, we also considered an alternative anion–radical stabilization scenario – one we initially proposed in ref. 7 – whereby the primary species (R^−^ and R˙) react with O_2_ in its triplet ground state to yield ROO^−^ and ROO˙, respectively. As it has been already reported, such oxygenated species are longer-lived than their R˙ counterparts,^36^ form rapidly after polymer bond breaking, and the reactions producing them are energetically favored, as evidenced by the calculated stabilization energies plotted in Fig. 5a and b (DFT and HF results).

**Fig. 6:**
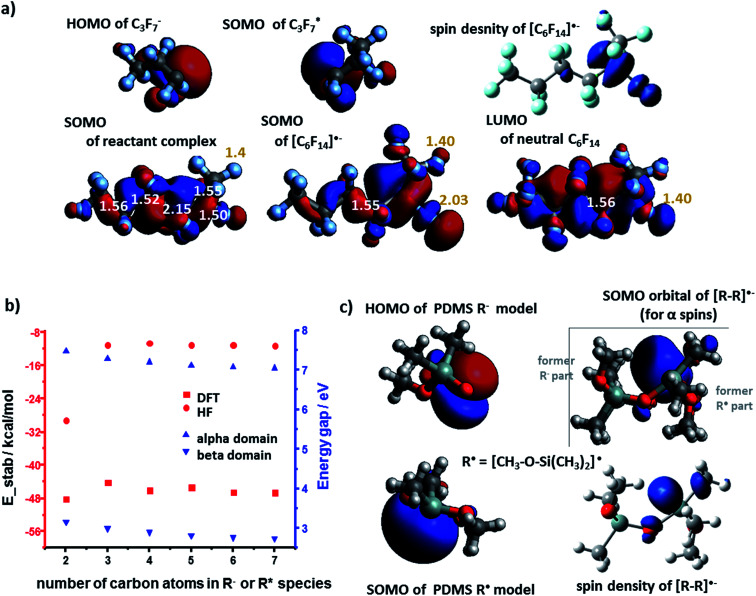
Anion–radical stabilization in (a and b) PTFE and (c) PDMS. (a) SOMO and HOMO orbitals of the C3 fragments making up the [C_6_F_14_]˙^−^ radical–anion adduct for which the spin density contour is also shown. LUMO of the uncharged C_6_F_14_ is included for comparison. In white font – C–C bond lengths in Å. In yellow font – C–F bond lengths in Å. (b) Blue markers: orbital energy gap (α and β domain), plotted as a function of the number of carbons in each of the interacting fragments. Red markers: total DFT and HF stabilization energies plotted as a function of fragment size. (c) PDMS HOMO NBO contour of R^−^ and SOMO NBO contour of R˙ for the 1-monomer model system. NBO bonding orbital contour for [R–R]˙^−^ alongside with spin density contour is also shown.

The analysis of PTFE system unveiled some subtle differences compared to the PE system. Upon bringing the R^−^ and R˙ into proximity, the half-filled antibonding C–C SOMO orbital is present within the so called pre-complex (a.k.a. reactant complex, RC, first row in Fig. 6a). Typically, such a complex exists only in the first stages of reaction, mostly due to weak van der Waals forces. For the true potential energy minimum, SOMO is an antibonding orbital of one of the (elongated) C–F bonds (second row in Fig. 6a). This electron density transfer suggests that [R–R]˙^−^ resembles more the [R–R˙⋯ F^−^] complex rather than a moiety held by a covalent-like bond – this finding agrees with previous theoretical studies of alkyl halides.^31^ The orbital gap for PTFE system is 7 eV for α spins and 2.5 eV for β spins (Fig. 6b) – that is slightly higher than in other systems we discussed. However, the total DFT energy of stabilization is favorable/negative, with absolute values between 44.2 kcal mol^−1^ to 48.2 kcal mol^−1^ (Fig. 6b). These values are less than for PE because highly-electronegative F atoms remove electron density from the newly formed C–C bond, thus weakening it.

Finally, for the PDMS case, the orbital (NBO) interaction analysis shows that the SOMO (for α spins) is not the simple antibonding orbital between the Si atom (from R˙) and O atom (from R^−^) but the orbital localized mostly on the Si atom – see Fig. 6c. This finding is in line with the spin density contour, indicating that electron density is transferred also onto nearby Si orbitals. More detailed analysis of orbital occupations and interactions (ESI, Section 5†) predicts total stabilization energies at 31.0 and 30.9 kcal mol^−1^ (absolute values) for the models based on the interaction between, respectively, the 1-monomer and the 2-monomer fragments (R^−^ with R˙). The energy gaps of the anion–radical adducts are 6.47 eV (1-monomer model) and 6.00 eV (2-monomer model).

## 4. Conclusions

In summary, the above theoretical studies substantiate our previous experimental findings^7^ that in electrified polymers radicals can stabilize nearby charged species of either polarity. While we have studied here only the model systems, this conclusion seems to be valid for different types of polymers albeit there are some subtle differences. Stabilization of cations is generally due to the interaction between unoccupied orbital of R^+^ (LUMO) with the SOMO orbital of nearby radical R˙, resulting in the creation of [R–R]˙^+^ species. Interaction of the R^−^ HOMO with R˙ SOMO is also allowed by orbital symmetries but the resulting thermodynamically stable anion–radicals might, in most cases, be temporary species, likely detaching an excessive electron (or anion) which is then stabilized in the nearby defects. In each case, a non-zero electron density between initially separated fragments is reported, reflecting formation of a new bond of fractional order.

While the present work was inspired by our experiments on contact-electrification of polymers, we suggest that the principle of charge-radical stabilization could be extended to the design of other molecular and macromolecular systems in which nearby – but not conjugated – charged and radicalic groups would interact. Could one then prepare derivatives of molecules such as TEMPO in which radical longevity could be increased by nearby charged groups? How would this influence the radical's reactivity? We believe such questions can provide fruitful avenues for future research on controlling chemical reactivity by flanking charges by radicals or radicals by charges.

## Supplementary Material

SC-008-C6SC02672A-s001
