# Supplementary material for: Theoretical basis for the stabilization of charges by radicals on electrified polymers
Source: Chem Sci. 2016 Nov 23;8(3):2025–32. doi: 10.1039/c6sc02672a (PMC5398273; doi:10.1039/c6sc02672a)
Supplement: SC-008-C6SC02672A-s001 [file SC-008-C6SC02672A-s001.pdf]

Electronic Supplementary Material (ESI) for Chemical Science.
This journal is © The Royal Society of Chemistry 2016

Supporting Information for Manuscript entitled "*Theoretical basis for the stabilization of charges by radicals on electrified polymers*" by Tomasz Mazur and Bartosz A. Grzybowski,

Section 1. Total energies, molecular orbital energies of optimized structures.

Table 1 shows total energies of the PE and PTFE model molecules in their optimized geometries. Data in Table 2 expands the PE model to include the secondary carbocation units. Table 3 lists DFT energies of the optimized PDMS models.

For PE and PTFE cases, additional Hartree-Fock (HF) calculations were also performed using the 6-311++G(d,p) basis set. This additional investigation was carried because previous DFT studies reported positive energy of the HOMO orbital for the anionic species (R^-) of PE, which is a known issue of the DFT methodology[29]. We additionally calculated by HF methods the HOMO (R^-) orbital energies (which had negative values), proving that no unbound electron was present in the model system. The detailed values of orbital energies of relevant orbitals are presented in Table 4 (PE and PTFE) and Table 5 (PDMS), alongside with their energy differences.

Table 1. Total energies of optimized R^{+}, R^{-}, $R^{\bullet}$, $[R\text{-}R]^{\bullet +}$ and $[R\text{-}R]^{\bullet -}$ for PE, PTFE and PDMS.

*all E in Ha		$R^{+}_{(primary)}$	R^{-}	$R^{\bullet}$	$[R\text{-}R]^{\bullet +}$	$[R\text{-}R]^{\bullet -}$
**Poliethylene (PE) DFT GD3 B3LYP/6-311++G(d,p)**	**C2**	-78.88455491	-79.17566460	-79.18632130	-158.13896160	-158.48756728
	**C3**	-118.23091937	-118.51171740	-118.51296820	-236.80657690	-237.14385462
	**C4**	-157.56439215	-157.83675430	-157.84009790	-315.46286780	-315.79934647
	**C5**	-196.89439964	-197.16489660	-197.16715260	-394.10583800	-394.45540550
	**C6**	-236.22294202	-236.49153480	-236.49422840	-472.75991860	-473.11111719
	**C7**	-275.55080780	-275.80490020	-275.82131260	-551.41302590	-551.76668314
**Poliethylene (PE) HF 6-311++G(d,p)**	**C2**	-78.33525909	-78.55418820	-78.62052350	-156.99987080	-157.29588970
	**C3**	-117.39246609	-117.60260950	-117.66456940	-235.09320810	-235.38464030
	**C4**	-156.43669553	-156.64713890	-156.70881710	-313.18442160	-313.47193960
	**C5**	-195.48807647	-195.69146111	-195.75292868	-391.2733663	-391.56044479
	**C6**	-234.53339773	-234.73581763	-234.79704018	-469.36343190	-469.64585480
	**C7**	-273.57815795	-273.78003920	-273.84115064	-547.45162000	-547.73670191
**Politetrafluoroethylene (PTFE) DFT GD3 B3LYP/6-311++G(d,p)**	**C2**	-575.18683971	-575.61312300	-575.53871462	-1150.76045040	-1151.22862670
	**C3**	-813.05189450	-813.47929670	-813.40000990	-1626.49088060	-1626.94971481
	**C4**	-1050.91398379	-1051.34109370	-1051.25966260	-2102.21944648	-2102.67423072
	**C5**	-1288.77493785	-1289.20242420	-1289.11922980	-2577.94618948	-2578.39388634
	**C6**	-1526.63541903	-1527.06306040	-1526.97897720	-3053.67166869	-3054.11623896
	**C7**	-1764.49559176	-1764.92338190	-1764.83875080	-3529.39640333	-3529.83646430
**Politetrafluoroethylene (PTFE) HF 6-311++G(d,p)**	**C2**	-572.70649712	-573.07109635	-573.04675243	-1145.74537305	-1146.16447013
	**C3**	-809.51765830	-809.88307620	-809.85483090	-1619.36524745	-1619.75551428
	**C4**	-1046.32632415	-1046.69088200	-1046.66085963	-2092.98029968	-2093.36934200
	**C5**	-1283.13346130	-1283.49807680	-1283.46681310	-2566.59112477	-2566.98254217
	**C6**	-1519.94016800	-1520.30471480	-1520.27289060	-3040.20398385	-3040.59524000
	**C7**	-1756.74661240	-1757.11113330	-1757.07899460	-3513.81549360	-3514.20764403

Table 2. Total energies of optimized R^{+} and $[R\text{-}R]^{\bullet +}$ for PE for the secondary carbocation case.

*all E in Ha		$R^{+}_{(secondary)}$	$[R\text{-}R]^{\bullet +}$ from $R^{+}_{(secondary)}$
**Poliethylene (PE) DFT**	**C3**	-118.24740114	-236.81297183
	**C4**	-157.58073341	-315.47327234
	**C5**	-196.91281062	-394.13355752

**GD3 B3LYP/6-311++G(d,p)**	**C6**	-236.24296553	-472.79188428
	**C7**	-275.57134814	-551.44993709
**Poliethylene (PE) HF 6-311++G(d,p)**	**C3**	-117.41340495	-235.10361592
	**C4**	-156.46242921	-313.19428028
	**C5**	-195.50968879	-391.28483572
	**C6**	-234.55555578	-469.37428232
	**C7**	-273.60042395	-547.46316612

Table 3. Total energies of optimized R^+, R^-, and two types of $R^\bullet$ (Si- and O- terminated) for PDMS.

***all E in Ha**		**R^+**	**$R^\bullet$ (pair to R^+)**	**$[R\text{-}R]^{\bullet+}$**	**R^-**	**$R^\bullet$ (pair to R^-)**	**$[R\text{-}R]^{\bullet-}$**
**PDMS DFT (single point) GD3 B3LYP/6-311++G(d,p)**	**1-monomer model**	-484.3242284	-559.8150942	-1044.2457233	-559.9274075	-484.5488098	-1044.5256057
	**2-monomer model**	-929.0909939	-1004.69174	-1004.573093	-929.3031125	-1933.78276	-1934.044093

Table 4. Orbital energy gap ($\Delta\varepsilon$) between interacting frontier orbitals for $R^\bullet$, R^+ and R^- for PE and PTFE (canonical orbital energies). The lower the value of $\Delta\varepsilon$, the greater the orbital overlap. $\Delta\varepsilon_1 = |\varepsilon_{R+,LUMO} - \varepsilon_{R\bullet,SOMO(\alpha)}|$, $\Delta\varepsilon_{2\alpha} = |\varepsilon_{R-,HOMO(\alpha)} - \varepsilon_{R\bullet,SOMO(\alpha)}|$, $\Delta\varepsilon_{2\beta} = |\varepsilon_{R-,HOMO(\beta)} - \varepsilon_{R\bullet,SOMO(\beta)}|$.

***all E in eV**		**R^+ LUMO**	**R^- HOMO (α and β)**	**R• SOMO (α)**	**R• LUMO (β)**	**$\Delta\varepsilon_1$**	**$\Delta\varepsilon_{2\alpha}$**	**$\Delta\varepsilon_{2\beta}$**
**Poliethylene (PE) DFT GD3 B3LYP/6-311++G(d,p)**	**C2**	-8.10	1.89	-5.63	-1.64	2.47	7.52	3.53
	**C3**	-7.52	1.39	-5.61	-1.66	1.91	7.00	3.05
	**C4**	-7.08	1.59	-5.60	-1.64	1.48	7.19	3.23
	**C5**	-7.06	1.37	-5.58	-1.63	1.48	6.95	3.00
	**C6**	-6.96	1.43	-5.58	-1.63	1.38	7.01	3.06
	**C7**	-6.96	1.26	-5.58	-1.62	1.38	6.84	2.88
**Poliethylene (PE) HF 6-311++G(d,p)**	**C2**	-4.09	-0.48	-9.56	1.25	5.47	9.08	1.73
	**C3**	-3.04	-0.62	-9.52	1.23	6.49	8.90	1.85
	**C4**	-3.96	-0.68	-9.51	1.23	5.55	8.83	1.91

	**C5**	-2.82	-0.71	-9.50	1.22	6.68	8.79	1.94
	**C6**	-2.74	-0.73	-9.49	1.21	6.76	8.76	1.94
	**C7**	-2.70	-0.74	-9.49	1.22	6.79	8.76	1.22
**Politetrafluoroethylene (PTFE) DFT GD3 B3LYP/6-311++G(d,p)**	**C2**	-11.59	-0.41	-7.89	-3.57	3.70	7.48	3.16
	**C3**	-11.50	-0.60	-7.90	-3.61	3.60	7.29	3.00
	**C4**	-11.30	-0.68	-7.88	-3.59	3.41	7.20	2.90
	**C5**	-11.20	-0.76	-7.88	-3.58	3.32	7.12	2.82
	**C6**	-11.16	-0.80	-7.88	-3.58	3.28	7.08	2.77
	**C7**	-11.14	-0.83	-7.89	-3.58	3.25	7.05	2.75
**Politetrafluoroethylene (PTFE) HF 6-311++G(d,p)**	**C2**	-6.25	-3.81	-12.78	1.43	6.53	8.97	5.24
	**C3**	-6.29	-3.93	-12.81	1.48	6.52	8.88	5.41
	**C4**	-6.10	-4.00	-12.79	1.51	6.69	8.80	5.51
	**C5**	-6.00	-4.04	-12.79	1.51	6.79	8.75	5.55
	**C6**	-5.97	-4.07	-12.79	1.51	6.82	8.73	5.57
	**C7**	-5.95	-4.08	-12.79	1.51	6.84	8.71	5.59

Table 5. Orbital energy gap ($\Delta\varepsilon$) between interacting frontier orbitals for $R^{\bullet}$, R^{+} and R^{-} for PDMS (NBO orbital energies). The lower the value of $\Delta\varepsilon$, the greater the overlap. $\Delta\varepsilon_1 = |\varepsilon_{\mathbf{R+},\mathrm{LUMO}} - \varepsilon_{\mathbf{R\bullet},\mathrm{SOMO}(\alpha)}|$, $\Delta\varepsilon_2 = |\, \varepsilon_{\mathbf{R-},\mathrm{HOMO}} - \varepsilon_{\mathbf{R\bullet},\mathrm{SOMO}(\alpha)}|$.

***all E in eV**		**R^+ LUMO**	**R• (O-terminated) SOMO (α)**	**$\Delta\varepsilon_1$**	**R^- HOMO**	**R• (Si- terminated) SOMO (α)**	**$\Delta\varepsilon_2$**
**PDMS DFT (single point) GD3 B3LYP/6-311++G(d,p)**	**1-monomer model**	-5.79	-8.34	-2.55	-0.72	-7.19	-6.47
	**2-monomer model**	-5.12	-8.56	-3.43	-0.99	-6.92	-6.00

Table 6. Stabilization energies (D3 + B3LYP/6-311++G(d,p)) due to van der Waals interactions between two neutral molecules (2 x C_3H_8 – “C3 model” and 2 x C_6H_{14} – “C6 model”) in both parallel and “head-to-head” orientations.”

Model	E of monomer / Ha	E of dimer (parallel orientation) / Ha	E of stabilization (parallel orientation) / kcal/mol	E of dimer (head-to-head orientation) / Ha	E of stabilization (head-to-head orientation) / kcal/mol
**C3**	-119,1854624	-238,3709248	-1,42	-238,3737215	-3,18
**C6**	-237,1666926	-474,3384463	-1,75	-474,3347515	-0,86

Section 2. Illustrative examples of conformational analyses.

Figure S1 shows examples of energy (D3-UB3LYP/3-21G) contour maps for PE C3 and C7 model systems. The optimal parameters established in these initial calculations are indicated in the insets and are subsequently used for higher-level geometry optimization with D3-UB3LYP/6-311++G(d,p).

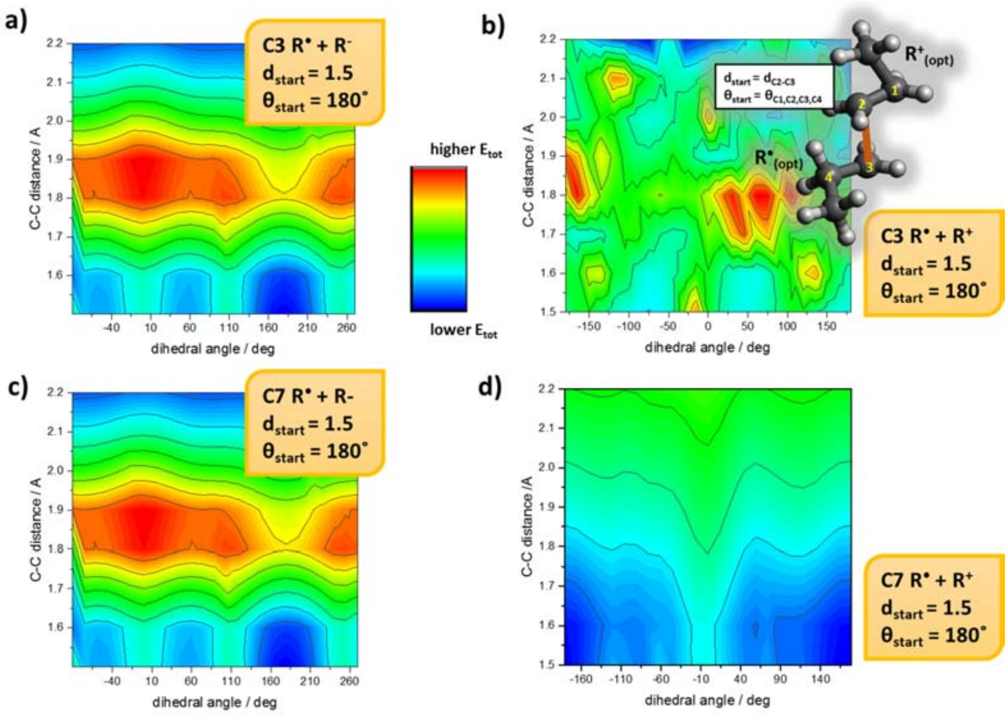

Figure S1. Examples of initial conformational analyses for PE fragments **(a)** energies of a C3 carboanion interacting with a C3 radical in different mutual orientations. Analogous plots for **(b)** C3 carbocation interacting with a C3 radical, **(c)** C7 carboanion interacting with a C7 radical, and **(d)** C7 carbocation interacting with a C7 radical. Insets have the values of parameters corresponding to energy minima. Calculations were performed using DFT (D3-UB3LYP/6-31G).

Section 3. Effects of PE chain branching.

Calculations on the influence of carbon chain branching (mimicking real-life polymer structure) on system's stability were performed for select/representative systems. Specifically, we chose four molecules for the 2-branched models and three molecules for the 3-branched models (see images in Figures S2a,b explaining the nomenclature). All the geometries of R^+, R^- and $R^•$ were optimized, using as starting points structures based on the ones presented in Figures S2a and S2b. In case of optimized cations, rearrangement from staring primary to secondary (2-branched modes) or tertiary (3-branched models) cations was observed. Additional single point calculations were performed for the starting geometry of cations. In each case, a branched $R^•$ model was compared with branched R^+ (of various geometries) or R^- models.

Based on the analysis of the results presented in Figure S2c, cation rearrangement causes the lowering of the molecular energy gap (by around 2eV) between SOMO ($R^•$) and LUMO (R^+) – at the same time strengthening these orbitals interactions. Additional carbon chain branches influence the relevant SOMO ($R^•$) and LUMO (R^+ in optimized geometry) orbital energies such that the energy gap is lowered by approximately 0.4eV (compare results of C2_2_X with C2_3_X in Figure S2c). The elongation of the carbon chain within each branch lowers the energy gap by another 0.9 eV (difference between C2_2_1 and C2_2_4) or 0.4 eV (difference between C2_3_2 and C2_3_4). In the anion-radical case, the energy gap between SOMO ($R^•$) and HOMO (R^-) is almost not influenced by branching or elongation (see Figure S2d).

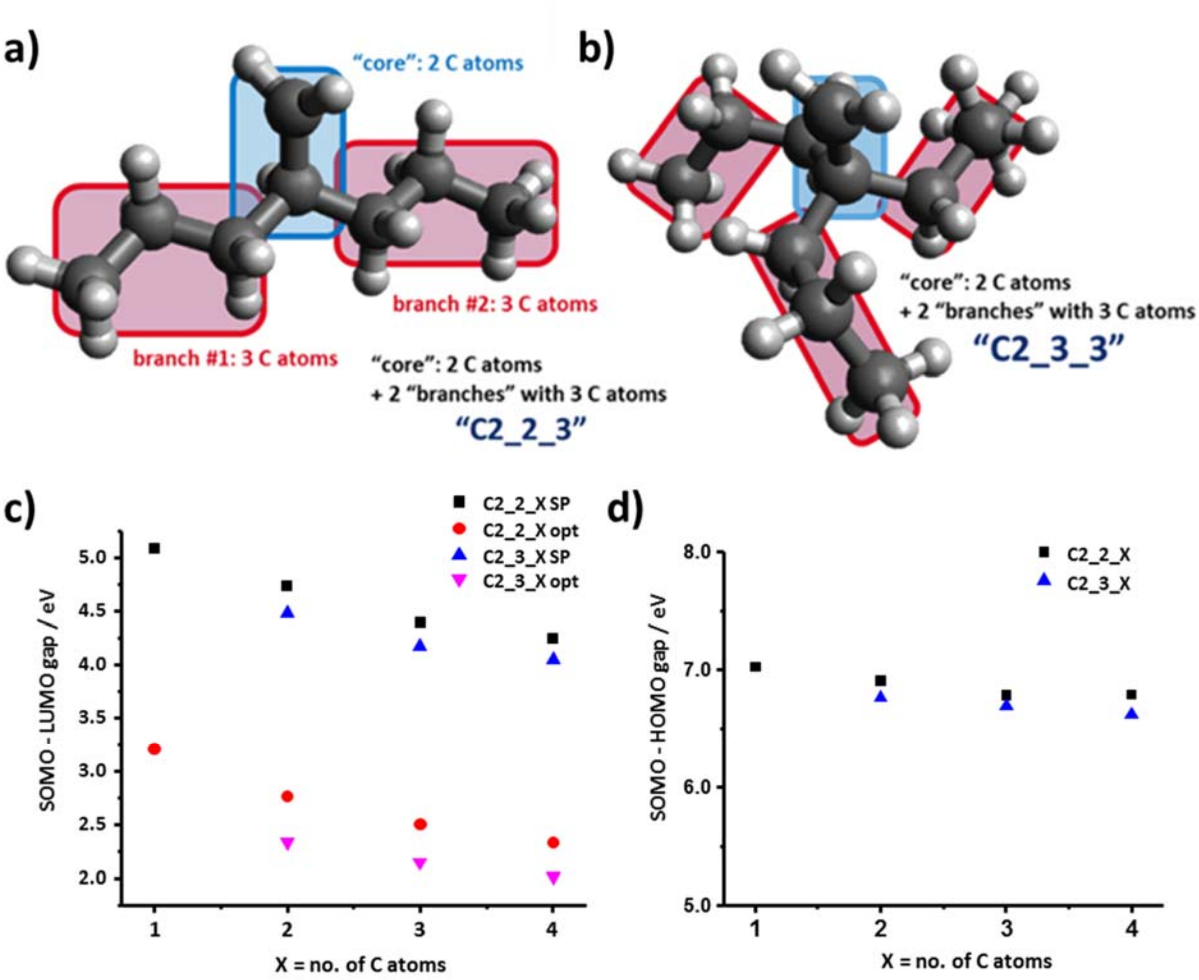

Figure S2. Examples of optimized geometries of radicals for the **(a)** 2- and **(b)** 3-branched species, alongside with short explanation of the nomenclature. Influence of branching on the orbital energy gap between interacting orbitals in stabilization schemes for **(c)** cation-radical – black squares (2-branched structures) and blue triangles (3-branched structures) for the constrained (single point calculations) primary cations; red circles and pink triangles for the optimized (tertiary) cations **(d)** anion-radical - black squares (2-branched structures) and blue triangles (3-branched structures).

Section 4. EPR studies of alkane cation-radicals.

The SOMO contours (examples shown in Figure S3a) of polyethylene's $[R\text{-}R]^{\bullet+}$ species calculated and discussed in the main text suggest that the unpaired electron is delocalized throughout the entire carbon chain. The existence of $[R\text{-}R]^{\bullet+}$ has been proven experimentally for several simple saturated hydrocarbons[22], and the related EPR spectra (see Figure S3b and c) indicated that the electron density is indeed σ-delocalized over the entire carbon chain. The hyperfine coupling between ^{1}H ($I(^{1}H)=1/2$) atoms connected to the same carbon is negligible (no spin density is present there) but the two terminal (most distant) H atoms are contributing to the EPR signal in a form of a triplet (1:2:1). With elongation of the carbon chain, the hyperfine coupling decreases, thus outer lines get closer to each other. The actual experiment was performed on the hydrocarbon molecules in $CFCl_2CF_2Cl$ matrix (in 77K). In our case, we modeled systems for which contact-electrification experiments were performed at room temperature – the results suggest that polymers can also stabilize species with σ-delocalized electrons.

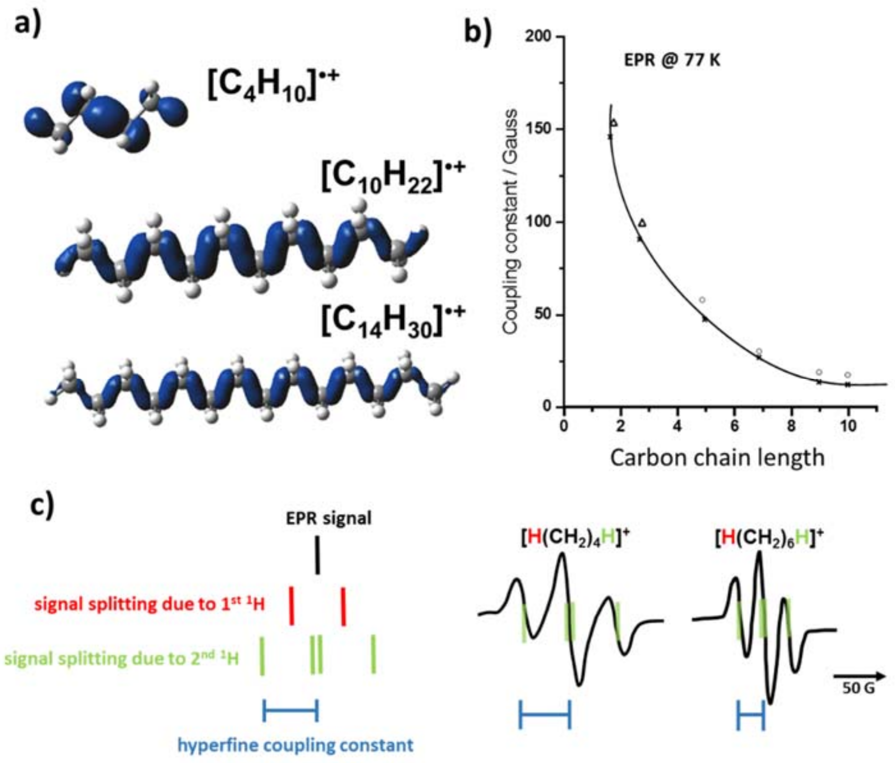

Figure S3. (a) Calculated sigma delocalization effect (density contour level = 0.004). Notice the diminishing spin density on terminal hydrogen atoms as the carbon chain is elongated. Picture adapted from reference 22. **(b)** EPR investigation of the cation radical species in $CFCl_2CF_2Cl$ matrix (at 77K) for several alkanes. **(c)** Theoretical model of the EPR signal splitting due to 1H interaction, alongside with sample EPR spectra.

Section 5. NBO analysis for (1monomer + 1 monomer) model of PDMS.

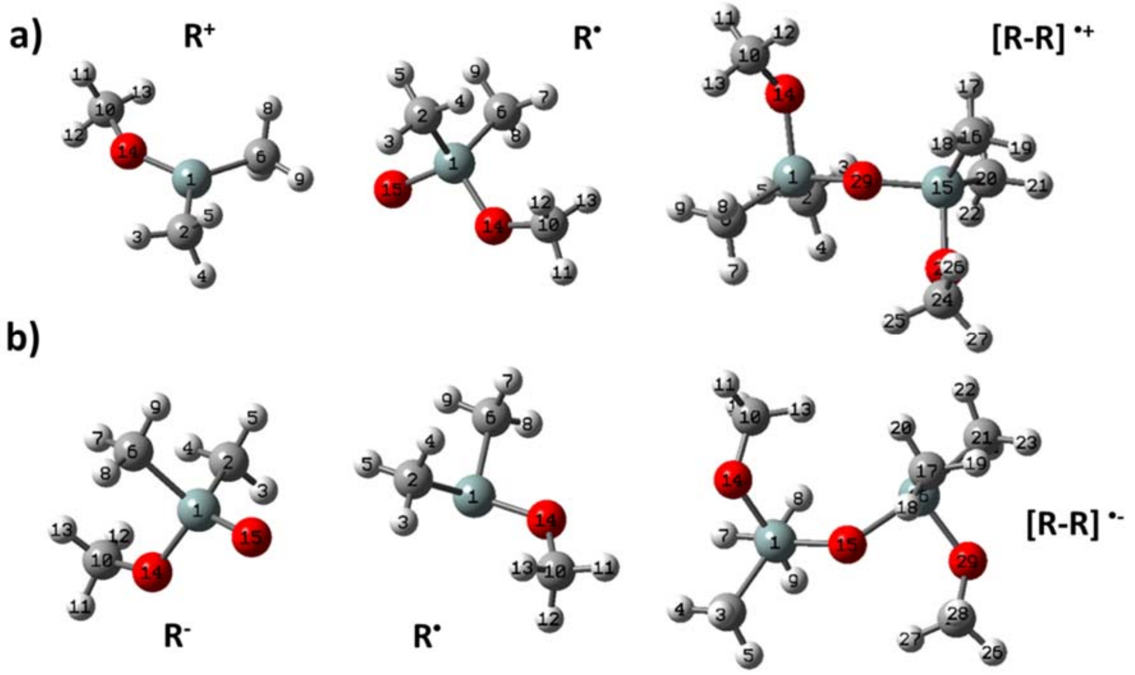

Figure S4. Atom labeling for the PDMS models (1 monomer-based) for the interaction of **(a)** cation with radical; **(b)** anion with radical.

In the discussion below, **BD** denotes a 2-center bonding orbital, **BD*** - 2-center antibonding orbital, **LP** stands for 1-center orbital (valence lone pair), and **X(Z)** denotes atom X, labeled by number Z.

For the PDMS $[R\text{-}R]^{\bullet+}$ case, we conducted NBO analysis. In both R^+ and $R^\bullet$ we identified interacting orbitals that are responsible for the resulting spin density.

a) R^+ electronic structure. The LUMO α orbital of the R^+ species (see Figure S4a for atom labelling) is the 27α orbital – BD* Si(1) - O(14), meaning the antibonding orbital between Si atom

labelled with number 1 and the O atom numbered 14. Its energy is -0.21266 Ha and it is almost fully unoccupied (residual occupation = 0.03606). This orbital consists in 90.39% of Si(1) *p*-type orbitals and in 9.61% of O(14) *p*-type orbitals.

b) R$^{\bullet}$ (oxygen-terminated) electronic structure. The SOMO α orbital of the R$^{\bullet}$ species is the orbital 15α described as LP O(15) – that is, a lone pair orbital on the O atom with label number 15. Its energy is -0.32090 Ha and occupation is 0.96464. The orbital comprises 99.78% of *p*-type orbitals of the O(15) atom.

c) [R-R]$^{\bullet+}$ electronic structure. In the [R-R]$^{\bullet+}$ adduct the most relevant orbitals are:

(i) Orbital 29α, BD Si(1)- O(29) – bonding orbital between Si(1) and O(29) with energy equal to -0.88816 Ha and occupancy of 0.99165. This orbital comprises 12.96% of Si(1) *p*-type and 87.04% of O(29) *s* and *p*-type orbitals.

(ii) Orbital 27β, BD Si(1)- O(29), with energy of -0.87373 Ha and occupancy of 0.99131. It comprises 13.22% of Si(1) *p*-type orbitals and 86.78% O(29) *s* and *p*-type orbitals

We emphasize that, contrary to the PE and PTFE cases, both (α and β) BD Si(1)- O(29) spinorbitals in [R-R]$^{\bullet+}$, originating from the interaction between initial R$^{\bullet}$ and R^{+} fragments (i.e. 29α and 27β) are occupied. This is unusual, as the SOMO(R$^{\bullet}$) orbital is only occupied in one (accordingly to its name – singly occupied molecular orbital), alpha domain. Judging from the spin density contour (see main text, **Error! Reference source not found.**d), the β electron density (on orbital 27β) comes from the donation of the oxygen atoms present in the PDMS structure.

Similar analysis was done for PDMS [R-R]$^{\bullet-}$ case (atom labelling presented in Figure S4b):

a) R^- electronic structure. The HOMO α orbital of the R^- species, is the orbital 14α – LP O(15), meaning a lone pair orbital on O atom number 15. Its energy is -0.02647 Ha, occupation = 0.93177. The orbital is made of *s* and *p*-type orbitals of Si(1) silicon atom. This orbital consists in 99.95% of O(15) *p*-type orbitals.

b) $R^{•}$ (silicon-terminated) electronic structure. The SOMO α orbital of the $R^{•}$ species is the orbital 10α described as LP Si(1) – meaning, a lone pair orbital on the Si atom with label number 1. Its energy is -0.26416 Ha and occupation equals 0.97761. The orbital is made of *s* and *p*-type orbitals of the Si(1) silicon atom.

c) $[R\text{-}R]^{•-}$ electronic structure. In the $[R\text{-}R]^{•-}$ adduct the most interesting orbitals are:

(i) Orbital 25α, LP Si (16) – a lone pair orbital on Si(16) with energy equal to -0.05180 Ha and occupancy of 0.86290. This orbital influences the spin density contour mostly and shows where the additional electron density resides.

(ii) Orbital 40β, BD O(15)- Si(16), with energy of -0.35441 Ha and occupancy of 0.97648. It comprises 85.54 % of O(29) s and p-type orbitals and 14.46% of Si(16) orbitals.

In contrast to the simplified orbital interaction model for anion-radical pairs (cf. Figure 1d in the main text), the SOMO α electron is localized on orbital bound to Si(16), instead of being localized on the antibonding orbital (such as BD* O(15)- Si(16)) – this fact is confirmed by the contour of the spin density for the whole molecule.

Section 6. Benchmarks of energy of stabilization.

Additional stabilization energies at more accurate levels of theory were computed using: altered D3 + B3LYP/(6-311++G(d,p) with corrections from BSSE, complete basis set method CBS-QB3,

and Gaussian-2 (G2) method. These benchmarks are shown for the polyethylene (PE) and polytetrafluoroethylene (PTFE) cases in Figures S5 and S6. The results were calculated as zero-point-corrected energies at 0K. For very complex energy computations – CBS-QB3 and G2 – models up to 6 carbon atoms (C3 dimers) in the chain were calculated. Correction from BSSE in the DFT results yields stronger stabilization energy for both PE and PTFE and for both cation radicals and anion radicals. In case of complete basis set approach (CBS-QB3), the results differ from DFT results by at most 5 kcal/mol, whereas G2 benchmark shows that in some cases (PE anion radical species) DFT stabilization can be overestimated by almost 20 kcal/mol.

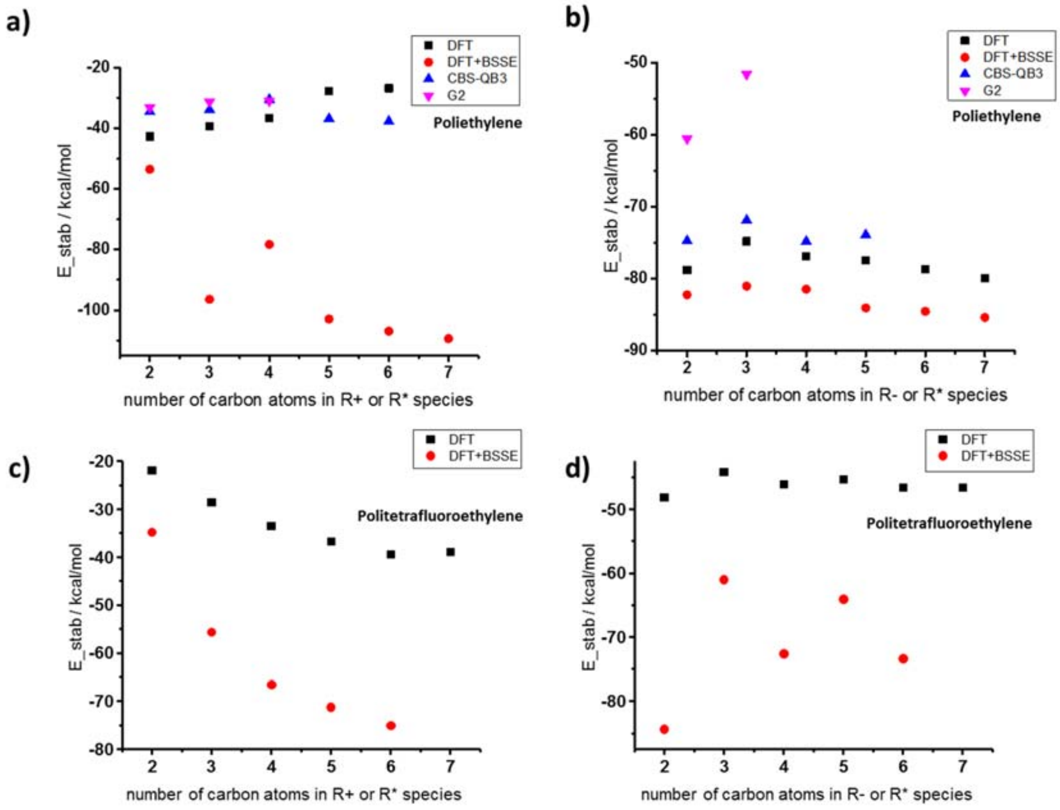

Figure S5. Stabilization energies (in kcal/mol) for **a)** cation-radical in PE, **b)** anion-radical in PE, **c)** cation-radical in PTFE, and **d)** anion-radical in PTFE model systems – as predicted by D3-

UB3LYP/ 6-311++G(d,p) **(black markers)**, D3-UB3LYP/ 6-311++G(d,p) with the inclusion of BSSE **(red markers)** , CBS-QB3 **(blue markers)**, and G2 **(pink markers)** levels of theory. CBS-QB3 and G2 results presented only for the PE case.

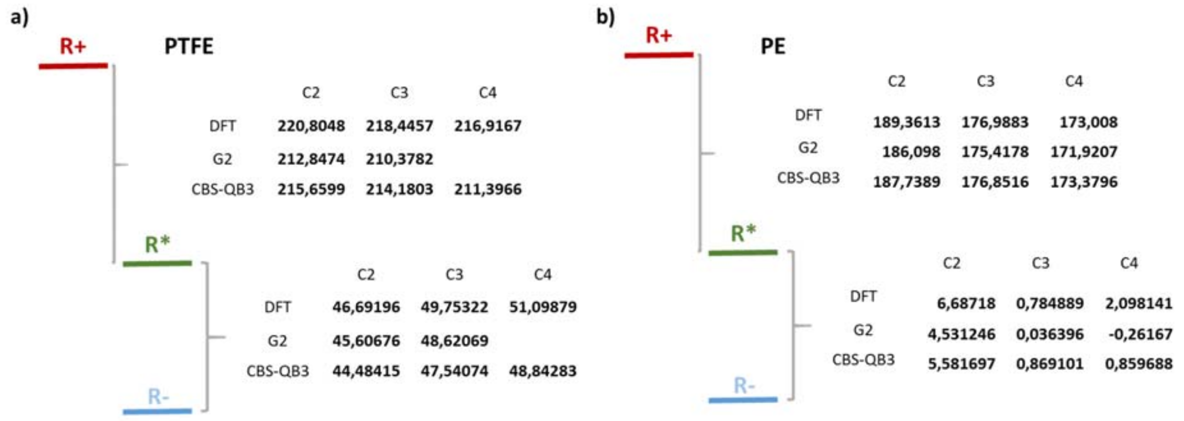

Figure S6. Relative energies (R* treated as 0 kcal/mol) of R^+ and R^- species for various methods (DFT = GD3 B3LYP/6-311++G(d,p), CBS-QB3, G2) for **a)** polytetrafluoroethylene (PTFE) and **b)** polyethylene (PE).

Section 7. Optimized coordinates for modeled systems.

Optimized geometries of relevant model systems are available as separate .xyz files. The directory and file structures are as follows:

```
PE_DFT (optimized geometries for B3LYP 6-311++G(d,p) + GD3)
      \C2
      files inside:
            C2a - anion
            C2r - radical
            C2c - cation
            C2ar - anion-radical
            C2cr - cation-radical

      \C3-C7 (CX)
```

```
      files inside:
            CXa - anion
            CXr - radical
            CXc1 - cation 1*
            CXc2 - cation 2*
            CXar - anion-radical
            CXc1r - cation-radical (from 1* cation)
            CXc2r - cation-radical (from 2* cation)

      \BRANCHING
            \C2_2_X (X: 1-4)
            files inside:
                  C2_2_Xa - anion
                  C2_2_Xr - radical
                  C2_2_Xc - cation (optimized)
                  SP_C2_2_Xc - cation (fixed geometry - single point
calculation)

            \C2_3_X (X: 2-4)
            files inside:
                  C2_3_Xa - anion
                  C2_3_Xr - radical
                  C2_3_Xc - cation (optimized)
                  SP_C2_3_Xc - cation (fixed geometry - single point
calculation)


      \ELONGATION_eps_1 (dielectric constant ε = 1)
            \C2-C15 (CX)
            files inside:
                  CXa - anion
                  CXr - radical
                  CXc - cation

      \ELONGATION_eps_2p27 (dielectric constant ε = 2.27)
            \C2-C15 (CX)
            files inside:
                  CXa - anion
                  CXr - radical
                  CXc – cation

      \ELONGATION_eps_1 (dielectric constant ε = 1)
            \C8-C15 (CX)
            files inside:
                  CXa - anion
                  CXr - radical
```

```
                CXc – cation

      \OXYGENATES
            \ROO
            files inside:
                  CXaOO (X: 2-7) - oxygenated anion
            \AOO
            files inside:
                  CXrOO (X: 2-7) - oxygenated radical
```

PE_HF (optimized geometries for HF 6-311++G(d,p))

```
      \C2
      files inside:
            C2a - anion
            C2r – radical
            C2c - cation
            C2ar - anion-radical
            C2cr - cation-radical

      \C3-C7 (CX)
      files inside:
            CXa - anion
            CXr - radical
            CXc1 - cation 1*
            CXc2 - cation 2*
            CXar - anion-radical
            CXc1r - cation-radical (from 1* cation)
            CXc2r - cation-radical (from 2* cation)

      \OXYGENATES
            \ROO
            files inside:
                  CXaOO (X: 2-7) - oxygenated anion
            \AOO
            files inside:
                  CXrOO (X: 2-7) - oxygenated radical
```

PTFE_DFT (optimized geometries for B3LYP 6-311++G(d,p) + GD3)

```
      \C2-C7 (CX)
      files inside:
            CXa - anion
            CXr - radical
            CXc1 - cation 1*
```

CXar - anion-radical
CXarRC - anion-radical (reactant complex)
CXc1r - cation-radical (from 1* cation)

PTFE_HF (optimized geometries for HF 6-311++G(d,p))
C2-C7 (CX)
files inside:
CXa - anion
CXr - radical
CXc1 - cation 1*
CXar - anion-radical
CXc1r - cation-radical (from 1* cation)

PDMS (optimized geometries for B3LYP 6-311++G(d,p) + GD3)
X_monomer (X: 1-2)
files inside:
SiX_r1 - radical (O-terminated)
SiX_c1 - cation (Si-terminated)
SiX_c1r1 - cation-radical
SiX_r2 - radical (Si-terminated)
SiX_a2 - anion (O-terminated)
SiX_a2r2 - anion-radical
